# Carcinogenesis and Prognostic Utility of Arginine Methylation-Related Genes in Hepatocellular Cancer

**DOI:** 10.3390/cimb45120591

**Published:** 2023-11-24

**Authors:** Waleed Ali, Weirui Xiao, Henry Hoang, Vincent Cali, Andre Kajdacsy-Balla

**Affiliations:** 1 Albert Einstein College of Medicine, The Bronx, NY 10461, USA; waleed.ali@einsteinmed.edu (W.A.); weirui.xiao@einsteinmed.edu (W.X.); henry.hoang@einsteinmed.edu (H.H.); vincent.cali@einsteinmed.edu (V.C.); 2 Department of Pathology, College of Medicine, University of Illinois at Chicago, Chicago, IL 60612, USA

**Keywords:** arginine methylation, hepatocellular carcinoma, TCGA, PRMT5, in silico

## Abstract

Protein arginine methylation is among the most important post-translational modifications and has been studied in cancers such as those of the lung and breast. However, comparatively less has been investigated regarding hepatocellular carcinoma, with an annual incidence of almost one million cases. Through using in silico methods, this study examined arginine methylation-related gene expression and methylation levels, and alongside network and enrichment analysis attempted to find how said genes can drive tumorigenesis and offer possible therapeutic targets. We found a robust relationship among the selected methylation genes, with ⅞ showing prognostic value regarding overall survival, and a medley of non-arginine methylation pathways also being highlighted through the aforementioned analysis. This study furthers our knowledge of the methylation and expression patterns of arginine histone methylation-related genes, offering jumping points for further wet-lab studies.

## 1. Introduction

Protein arginine methylation is one of the most biologically relevant forms of post-translational modification in which a covalent bond is formed with the addition of a methyl group to arginine residues [1]. This process is catalyzed with protein arginine methyltransferases (PRMTs). PRMTs function by covalently linking one or two methyl groups to a guanidino nitrogen of the synthesized protein’s arginine residues [2]. This process has been implicated in a vast array of cellular functions including transcription regulation, RNA transcription, DNA repair, and signal transduction [3]. Given their ubiquitous role, involvement of PRMTs in the regulation of the cell cycle remains a target for cancer studies.

PRMT5 has been shown to promote the degradation of cyclin E which serves to arrest cell-cycle proliferation [4]. Conversely, the methylation of CDK9 with PRMT1 accelerates transcriptional activity and thus cell-cycler progression [5]. P53, one of the most studied tumor suppressor genes, is also affected by PRMTs [6]. Specifically, PRMT5-induced methylation of P53 serves to impair its transcription and leads to downstream inhibition of P53 target genes; this process impairs inherent cell controls on cell-cycle arrest and apoptosis [7]. Consequentially, the overexpression of PRMT1s has been implicated in several cancers including breast, prostate, lung, and melanoma [8]. Additionally, PRMT5 has been shown to be overexpressed in ovarian cancer [9]. The sub-variants of PRMTs and their demonstrated effects on cancer progression demonstrate that the further investigation of the arginine methylation process in cancer is necessary.

A relative dearth of information regarding the arginine methylation process exists regarding liver cancer, which is a growing global healthcare problem necessitating increased attention worldwide. It is estimated that the annual incidence of liver cancer will surpass one million cases by 2025, with a staggering 841,080 new cases reported in 2018 alone [10]. Liver cancer ranks as the sixth most common cancer worldwide and the fourth leading cause of cancer-related deaths. Hepatocellular carcinoma (HCC) is a malignancy that arises from hepatocytes, the primary functional cells of the liver. HCC is the most common form of primary liver cancer, accounting for approximately 90% of liver cancer cases. Despite significant therapeutic advancements, HCC exhibits an exceptionally poor prognosis, as evidenced by a 5-year survival rate ranging between 15% and 38% in the United States and Asia. This is primarily due to delayed diagnosis, chemotherapy resistance, and recurrent metastasis [11]. Furthermore, it is important to note that the mechanism underlying tumor development in hepatocytes may exhibit unique characteristics compared to other tissues. Hepatocytes have an overall decreased rate of division, specifically because chromosomal duplication can occur even without concomitant cellular division.

With the advent of new genomic platforms such as the Cancer Genome Atlas (TCGA) and other data mining sites, in silico research offers a useful avenue to examine how arginine methylation affects the development and growth of liver cancer. In our study, we examined arginine methylation-related protein expression in HCC cases (stratified based on survival and staging) and conducted network analyses to see how arginine methylation may affect tumor development and growth.

## 2. Materials and Methods

### 2.1. Selection of the Genes

The genes selected for this study were obtained from the gene ontology accession GO:0034969 covering “histone arginine methylation”. The following genes were utilized in this paper’s analysis: *PRMT5* (protein arginine methyltransferase 5), *PRDM4* (PR/SET domain 4), *CLNS1A* (chloride nucleotide-sensitive channel 1A), *METTL23* (methyltransferase like 23), *PRMT1* (protein arginine methyltransferase 1), *COPRS* (coordinator of *PRMT5* and differentiation stimulator), *PRDM14* (PR/SET domain 14), and *WDR77* (WD repeat domain 77).

### 2.2. KM Plots

The KM-plotter (http://kmplot.com/analysis/) is an internet-based tool utilized in this study to evaluate the connection between predicted targets and survival in a particular cancer. This study analyzed the overall survival (OS) of hepatocellular cancers taken from the TCGA dataset in relation to selected genes associated with arginine methylation. For all genes listed, the same “Pan-Cancer” analysis was conducted. When conducting the KM analysis, an auto cutoff was selected with no restriction in place based on subtype (stage, gender, etc.) or restriction based on cellular content. Data were accessed in August of 2023. The results were examined using a Bonferroni corrected logrank *p* value (*p* ≤ 0.00625).

### 2.3. Methylation Analysis

The DNA methylation data obtained from the Illumina Infinium HumanMethylation450 BeadChip platform were employed in this study. The data were matched to both primary tumors and normal liver tissue samples using the UALCAN database (http://ualcan.path.uab.edu/). The average methylation value (beta) for the various arginine methylation-related genes, determined based on CpG sites, was compared using Welch’s *t*-test.

### 2.4. Expression Analysis

Through the Gene Set Cancer Analysis (GSCA) platform, an ANOVA test was completed comparing log2median centered mRNA expression levels for pathological staging of HCC groups I–IV. Boxplots were generated for each gene, with *p* < 0.05 being the cutoff for within-group comparison.

### 2.5. Network-Enrichment Analysis

GeneMANIA (http://www.genemania.org) is a platform that enables the visualization of various biological interactions at different levels (such as co-expression, co-localization, and domain similarity) for a specific gene or genes. Through GeneMANIA, a gene–gene interaction network was generated for the network analysis of arginine methylation-related genes. Additional work was carried out using R (https://www.r-project.org/) and the Cytoscape platform (https://cytoscape.org/). The genes highlighted in the network analysis and the base arginine methylation genes then underwent enrichment analysis to highlight which biological processes they may have a role in. Annotations such as those under the Gene Ontology (GO) and Kyoto Encyclopedia of Genes and Genomes (KEGG) were analyzed through the Metascape (http://metascape.org) platform. Analysis was also conducted to see if any transcription factors had targets that were significantly enriched in the gene list. A minimum overlap of 3 genes and enrichment of 1.5 was set. A *p* < 0.05 was considered significant.

## 3. Results

### 3.1. Certain Arginine Methylation-Related Proteins Are Differentialy Expressed Based on Survival and Staging

**Figure 1:**
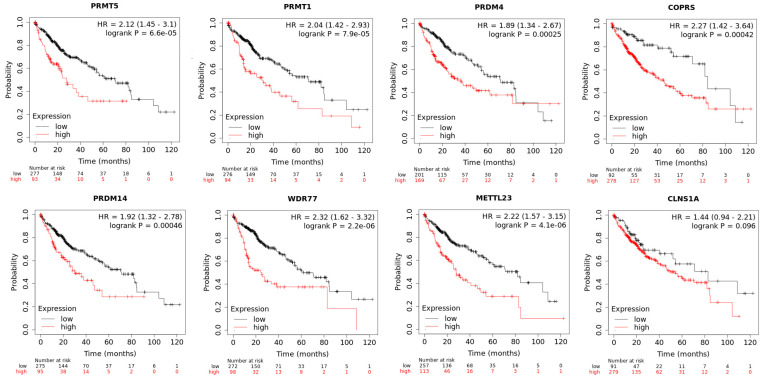
Kaplan–Meier plots based on mRNA expression of the selected arginine methylation genes. Each plot shows the numbers of pts in “high” versus “low” alongside associated hazard ratio (HR) within 95% CI.

Through the KMPlotter platform, the eight aforementioned genes’ mRNA expressions were analyzed for a relationship with overall survival in the TCGA dataset, as seen in Figure 1. All KM plots highlight a worse prognosis with higher levels. Out of the genes, all but one (*CLNS1A*) met the Bonferroni cutoff (*p* ≤ 0.00625): *PRMT5* (*p* = 6.6 × 10^−5^, HR = 2.12) *PRDM4* (0.00025, HR = 1.89), *METTL23* (*p* = 4.1 × 10^−6^, HR = 2.22), *PRMT1* (*p* = 7.9 × 10^−5^, HR = 2.04) *COPRS* (*p* = 0.00042, HR = 2.27), *PRDM14* (*p* = 0.00046, HR = 1.92), and *WDR77* (*p* = 2.2 × 10^−6^, HR = 2.32).

**Figure 2:**
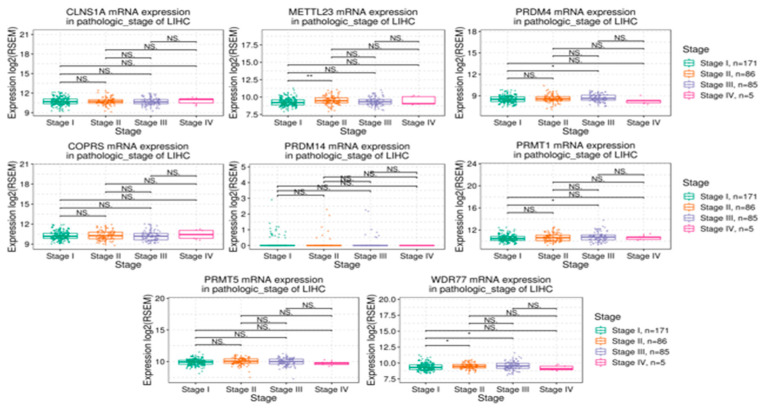
Dotplots showing mRNA expression levels (log2 median) based on pathologic staging taken from the TCGA datasets. For stages I, II, II, and IV, there were 171, 86, 85, and 5 cases, respectively, with all levels shown to be different based on ANOVA indicated by an * (*p* < 0.05) or ** (*p* < 0.01) NS = Not Significant.

Boxplots were generated showing log2-median mRNA levels for the various methylation-related proteins taken from the TCGA dataset, as separated by LIHC (Liver Hepatocellular Carcinoma) staging, as seen in Figure 2. ANOVA with a cutoff of <0.05 was used. Significant expression differences were distinguished with *. No significant changes in expression of *CLNS1A*, *COPRS*, *PRDM14*, and *PRMT5* were found between various pathologic stages of LIHC. Significant differences in expression of *METTLE23* were seen between stages I and II (*p* < 0.01) as well as for *WDR77* (*p* < 0.05). For *WDR77* and *PRMT1*, expression was also different between pathologic stages I and III (*p* < 0.05). Only in *PRDM4* was a significant difference between Stage I and Stage IV observed (*p* < 0.05).

### 3.2. DNA Methylation Patterns for the Arginine Methylation Genes Differ Significantly among Matched Normal and Primary Hepatocellular Cancers

**Figure 3:**
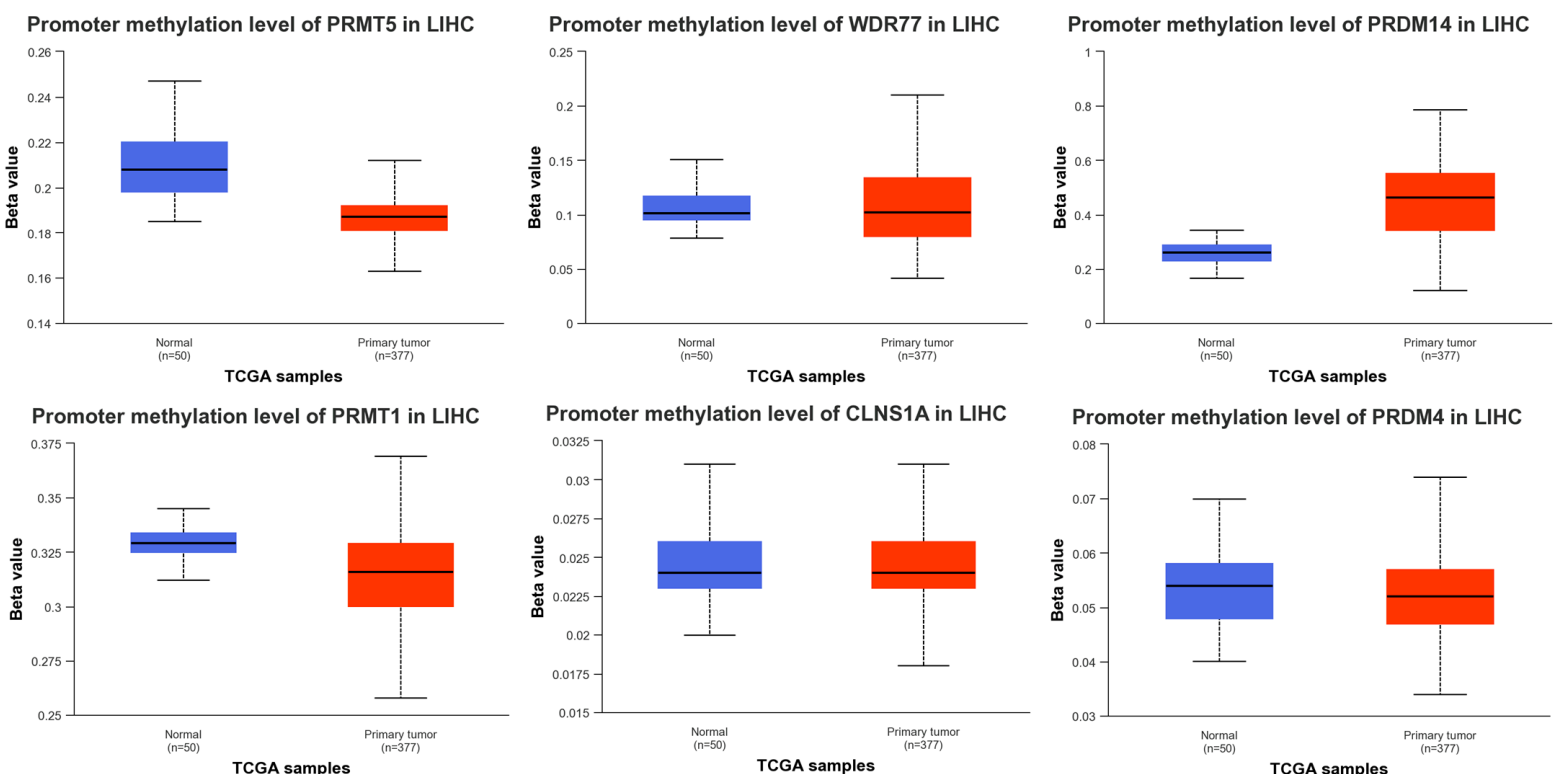
Boxplots showing methylation status of arginine methylation genes in normal vs. tumor tissue, with beta/methylation value on the Y-axis.

Boxplots depicting the beta-values (measure of methylation trends) were generated through the UALCAN interface using TCGA tagged data, as seen in Figure 3. *METTL23* data were not obtained, and accordingly not included in the analysis beta values of 0.25–0.3 which depict hypo-methylated, and 0.5–0.7 were hyper-methylated genes. While none of the genes in question showed to be directly hypermethylated, significant differences (*p* < 0.001) in some of the methylation patterns were seen. Comparison of beta values between the normal (blue) and the primary tumor (red), showed statistically significant differences for the genes *PMRT5*, *PRMT1*, and *PRDM14* (*p =* 9.98 × 10^−12^, 7.88 × 10^−15^, and 1.62 × 10^−12^, respectively).

### 3.3. Network and Enrichment Analysis Highlights a Myriad of Genes and Pathways through Which Arginine Methylation Can Impact Cancer Progression

**Figure 4:**
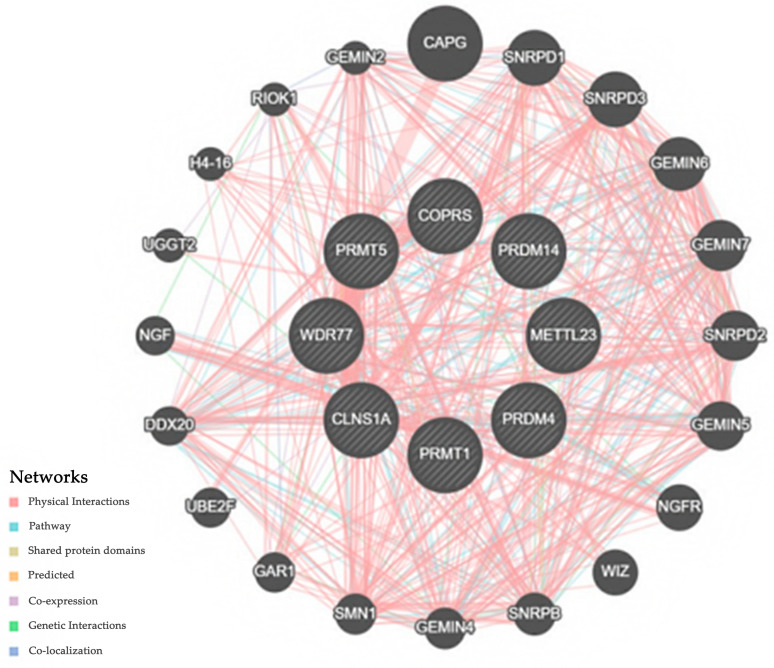
Network analysis of arginine methylation-related genes. Analysis was computed based on predicted and physical interactions among other genetic features such as overlapping domains.

Network analysis, and subsequently an enrichment analysis, was conducted on the genes located at the “outer rim” of each network, as seen in Figure 4. This analysis aimed to identify enriched biological pathways or functional categories within this gene set. The findings implicated various pathways, including spliceosomal snRNP assembly, SC1-based cell-cycle regulation, and other pathways associated with histone arginine methylation, which was expected given the initial focus on arginine methylation in the GO analysis.

**Figure 5:**
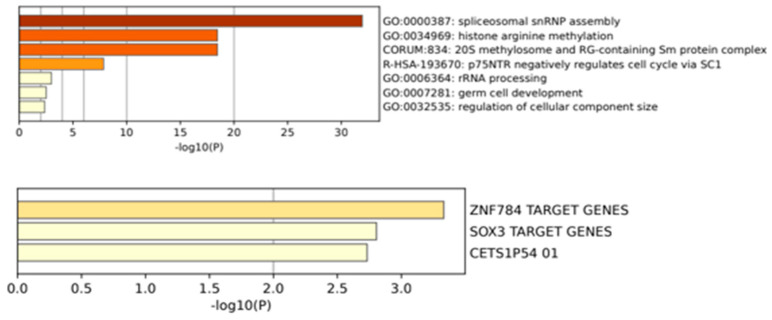
Enrichment analysis of the aforementioned genes found on the outside rim of the network analysis to see biological processes overrepresented with the genes highlighted. Additionally, the same subset of genes were analyzed to see any significantly enriched TFs which mediate their expression.

Moreover, as seen in Figure 5, the enrichment analysis revealed that the genes identified in the network analysis were significantly more likely to be targeted by two specific transcription factors, namely ZNF784 and SOX3.

## 4. Discussion

Overall, we see a remarkably robust relationship between the selected methylation genes and outcomes, even if that relationship is not necessarily reflected in the expression as per pathological stage. Of the studied genes, only CSNS1A failed to show a significant relationship between expression and overall survival. This significance, in terms of methylation genes and prognosis, mimics what is seen in the literature for other kinds of cancers. *METTL23* has been validated as a prognostic marker in prostate cancer [12], and the coactivation properties of *WDR77* on the androgen receptor have been linked to ovarian cancer. In contrast, CORP5 does not appear in the literature regarding discussion of its use as a possible prognostic factor; however, the strong relationship CORP5 has with PRMT5 as a co-binding factor may explain its significant relationship to the outcome.

While not implicated in this study regarding hepatocellular cancer, the *CLNS1A* gene has been implicated in other cancers, especially breast cancer. Amplification of *CLNS1A* was linked to worse outcomes in luminal breast-cancer tumors, possibly by helping to recruit proteins to the PRMT5 complex [13,14]. Arguably, the most research into how PRMT5 modulates cancer progression has been achieved in breast cancer research. Beyond binding to histone substrates on c-Myc and cyclinD1, they are well-established modulators of cell-cycle progression [15]; growth studies have also revealed PRMT5 causes alternative splicing in zinc-family proteins (namely ZNF326) [16,17]. This causes mRNA decay and a more oncogenic state. In hepatocellular carcinoma, PRMT5 has been linked to the increased half-life of SREBF1, an inducer of lipogenesis in the liver, which did not appear in the network analysis [18].

Methylation changes in hepatocellular carcinoma occur in hepatocellular carcinoma’s early stages, without cumulative changes as the tumor progresses to higher stages. Based on physical interactions, shared pathways, and motifs, we see a multitude of ways in which the highlighted genes can modulate survival outcomes. *CAPG*, a gelsolin protein family member which regulates actin filament remodeling, has been shown to be a prognostic marker in both breast and bone cancer [19,20]. Alongside PRMT5, SNRPDI regulates the cell cycle through the M checkpoint and has been tied to lung and breast cancer [21,22]. In hepatocellular cancer, SNRPDI has been linked to overexpression in the mTOR pathway [23].

One of the most well-studied cancer targets highlighted in our network analysis is the nerve growth factor receptor (NGFR). It has been linked to a myriad of cancers ranging from colorectal and pancreatic to NSCLC and paragangliomas, likely through its negative feedback of p53 [24,25,26,27]. The most represented family of proteins in our analysis are the gem nuclear organelle associated proteins (GEMIN; our analysis highlighted GEMIN2 and 4–7). Involved in the creation of small nuclear ribonucleoproteins (snRNPs) which help direct cellular pre-mRNAs, the GEMIN proteins have been linked to a variety of cancers through differential spliceosome activity among cancerous versus non-cancerous tissues. As a co-transcriptional process, epigenetic modifications secondary to methylation-expressed genes can affect the GEMIN proteins and may have a role in their differential expression in cancers, as per the literature [28,29].

We also observe an overrepresentation of genes regulated by the transcription factor SOX3 and ZNF784 from the network analysis. SOX3’s role in inducing epithelial-mesenchymal transformation has been well-established [30]. Increased migration secondary to SOX3 downregulation in gastric cancer has been shown, with the opposite shown in osteosarcoma in which downregulation of SOX3 decreased the snail/twist axis [31,32]. On the other hand, little has been studied regarding the possible oncogenic properties of ZNF784. However, ZNF as a family are among the most ubiquitous family of regulatory proteins. Examples include ZKSCAN3, which modulates colorectal tumorigenesis through VEGF, and ZFX, which promotes cell growth through the ERK-MAPK pathway [33,34]. Given the role other zinc finger proteins play, ZNF784 may be a suitable target for further analysis.

While this study makes progress in examining the methylation and expression patterns of arginine histone methylation-related genes, there are some caveats to these findings. These analyses are based on mRNA steady-state levels as a substitute for protein levels. The biologic activity of these mRNA may be additionally regulated with post-translational factors. This holds especially true for those genes which depend on secondary or tertiary factors for activity such as histone presentation. This can be a jumping point for further studies to directly examine the gene-expression relationship with prognosis and outcome at the protein or enzymatic level. In addition, these findings can guide further studies on prognostic factors for various categories of liver disease and treatment modalities which would not be fully accounted for with outcomes at the gene, mRNA, or protein level.

## 5. Conclusions

There is a robust relationship between the selected histone methylation genes and outcomes. Changes in methylation pattern occur in Stage I hepatocellular carcinoma but do not vary significantly between low-stage and high-stage tumors.

## Data Availability

Publicly available datasets were analyzed in this study. These data can be found at https://www.cancer.gov/ccg/research/genome-sequencing/tcga (accessed on 1 July 2023).
